# Maternal health policy environment and the relationship with service utilization in low- and middle-income countries

**DOI:** 10.7189/jogh.13.04025

**Published:** 2023-05-10

**Authors:** Andreea A Creanga, Martin AJ Dohlsten, Elizabeth K Stierman, Allisyn C Moran, Meighan Mary, Elizabeth Katwan, Blerta Maliqi

**Affiliations:** 1 Department of International Health, Johns Hopkins Bloomberg School of Public Health, Baltimore, Maryland, USA; 2 International Center for Maternal and Newborn Health, Johns Hopkins Bloomberg School of Public Health, Baltimore, Maryland, USA; 3 Department of Gynecology and Obstetrics, Johns Hopkins School of Medicine, Baltimore, Maryland, USA; 4 Department of Maternal, Newborn, Child, Adolescent Health and Ageing, World Health Organization, Geneva, Switzerland

## Abstract

**Background:**

The extent to which a favorable policy environment influences health care utilization and outcomes for pregnant and postpartum women is largely unknown. In this study, we aimed to describe the maternal health policy environment and examines its relationship with maternal health service utilization in low- and middle-income countries (LMICs).

**Methods:**

We used data from World Health Organization’s 2018-2019 sexual, reproductive, maternal, newborn, child, and adolescent health (SRMNCAH) policy survey linked with key contextual variables from global databases, as well as UNICEF data on antenatal care (ANC), institutional delivery, and postnatal care (PNC) utilization in 113 LIMCs. We grouped maternal health policy indicators into four categories – national supportive structures and standards, service access, clinical guidelines, and reporting and review systems. For each category and overall, we calculated summative scores accounting for available policy indicators in each country. We explored variations of policy indicators by World Bank income group using χ^2^ tests and fitted logistic regression models for ≥85% coverage for each of four or more antenatal care visits (ANC4+), institutional delivery, PNC for the mothers, and for all ANC4+, institutional delivery, and PNC for mothers, adjusting for policy scores and contextual variables.

**Results:**

The average scores for the four policy categories were as follows: 3 for national supportive structures and standards (score range = 0-4), 5.5 for service access (score range = 0-7), 6. for clinical guidelines (score range = 0-10), and 5.7 for reporting and review systems (score range = 0-7), for an average total policy score of 21.1 (score range = 0-28) across LMICs. After adjusting for country context variables, for each unit increase in the maternal health policy score, the odds of ANC4+>85% increased by 37% (95% confidence interval (CI) = 1.13-1.64) and the odds of all ANC4+, institutional deliveries and PNC>85% by 31% (95% CI = 1.07-1.60).

**Conclusions:**

Despite the availability of supportive structures and free maternity service access policies, there is a dire need for stronger policy support for clinical guidelines and practice regulations, as well as national reporting and review systems for maternal health. A more favorable policy environment for maternal health can improve adoption of evidence-based interventions and increase utilization of maternal health services in LMICs.

Safe and effective interventions exist for the prevention and treatment of the major causes of maternal mortality and morbidity [1]. To successfully adopt these evidence-based interventions, barriers that limit access to quality maternal health services must be identified, acknowledged, and addressed at all levels of the health system. Such barriers include proximal factors within the health system, but also distal factors like health governance and the policy environment for maternal health [2-4]. Despite widespread agreement that public policies impact population health everywhere in the world [5], the extent to which a favorable policy environment and specific maternal health policies and related structures influence health care utilization and outcomes for pregnant and postpartum women is largely unknown.

Understanding the role of maternal health policies is crucial for the Sustainable Development Goals (SDG). Globally, countries have committed to ending preventable maternal mortality and reduce the global maternal mortality ratio to <70 per 100 000 births, with the aim of no country having a ratio of more than twice the global average by 2030 [6]. Meeting this target will require average reductions of about three times the annual rate of reduction achieved during the Millennium Development Goals era [7]. Unfortunately, based on current progress, the world will fail to achieve the SDG goal at a cost of more than one million lives [8].

Efforts over the past two decades to monitor progress made in maternal survival uncovered deep inequities in outcomes as well as utilization of health services and quality of maternity care [9,10]. Most notably, the maternal mortality ratio declined by 38% between 2000 and 2017, with 94% of maternal deaths occurred in low- and lower-middle income countries [7]. Current global multi-partner initiatives such as the Strategies for Ending Preventable Maternal Mortality (EPMM) and Every Newborn Action Plan (ENAP) emphasize the need to increase the effective coverage of maternal health services, but also to address broader elements of health governance and policy to improve maternal health [11-13]. We aim to characterize the policy landscape for maternal health by examining the availability of national supportive structures and standards, service access policies, clinical guidelines, and reporting and review systems for maternal health, and to assess whether the availability of these national policies was associated with maternal health service utilization within low and middle-income countries (LMICs).

## METHODS

### Conceptual framework

**Figure 1:**
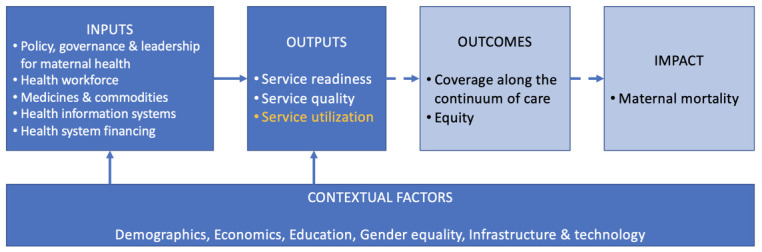
Conceptual Framework. Adapted from Singh et al. [14]. Inputs in line with WHO building blocks.

To guide our study, we developed an analytic framework adapted from Singh et al.’s [14] health systems and policy assessment framework that conceptualizes the World Health Organization (WHO) health systems building blocks and a country’s legislative framework for maternal health as inputs to the provision and utilization of maternal health services, the effective and equitable coverage of services along the continuum of care, and ultimately, maternal mortality (**Figure 1**). Given data availability and the many factors influencing effective and equitable coverage of interventions and maternal mortality, we focused on the relationship between policies and related implementation structures (e.g. coordination, clinical guidelines, reporting systems) for maternal health and utilization of key services in various countries: antenatal care (ANC), institutional delivery, and postnatal care (PNC) for the mother.

### Data sources and measures

We conducted a secondary analysis of cross-sectional data from the 113 LMICs that responded to the WHO sexual, reproductive, maternal, newborn, child, and adolescent health (SRMNCAH) policy survey in 2018-2019 [15]. This is the fifth and most recent such survey, revised to align with the SDGs and Global Strategy for Women’s, Children’s, and Adolescents’ Health (2016-2030) [16]. In relation to this survey, WHO developed an online platform to collect source documentation to allow validation of policy survey responses against national laws, policies, and guidelines [17].

We grouped maternal health policies and related structures assessed in the survey into four distinct categories and generated composite measures for each, calculating corresponding summative scores after assigning a score of 1 for each available policy indicator: a) national supportive structures and standards with four policy indicators (score range = 0-4), b) service access policies in the public health sector with seven policy indicators (score range = 0-7), c) national clinical guidelines and recommended practices with seven policy indicators (score range = 0-10) (to allow differentiation between partial (score of 1) and full (score of 2) counselling content in clinical guidelines for each of ANC, childbirth, and PNC), and d) national reporting and reviews with seven policy indicators (score range = 0-7). The full maternal health policy score ranged between 0 and 28, allowing for variation between and within country groups using World Bank 2018 income classification (low, lower-middle, and upper-middle income groups) [18]. We explored this variation by World Bank Income Group using χ^2^ tests for each policy indicator and James’ test for equal means for each of the four policy categories and overall, as it allows heterogeneous covariance matrices across by-groups.

We then identified other contextual factors hypothesized to influence adoption of maternal health policies, maternal health service utilization, and quality of care using several other data sources: the Worldwide Governance Indicators project [19] for a composite governance score for political stability and absence of violence, the WHO Global Health Workforce Statistics Database [20] for density of medical doctors per 10 000 population, the WHO Global Health Expenditure Database [21] for the domestic general government health expenditures per capita, the Population Reference Bureau [22] for the total fertility rates and the percentage of population living in urban areas, the World Bank’s World Development Indicators database [23] for the gender development index score, and the International Telecommunication Union database [24] for the percentage of the population with mobile cell phone subscriptions (Table S1 in the **Online Supplementary Document**). We chose these indicators based on their relevance and prominence after considering data availability across the 113 LMICs (e.g. high proportion of missing values for nurse density per 100 000 population, but not for doctor density per 100 000 population), and preliminary analyses finding high correlations with other predictors representing the same WHO health systems building block (e.g. seats for women in parliaments and political stability score). We linked the data for these indicators with the country-specific data in the SRMNCAH policy survey, after which we explored their variation by the World Bank income group classification using the abovementioned statistical tests.

The source of maternal health service utilization data, specifically 4+ ANC visits, institutional delivery, and PNC for mothers, was UNICEF [25]. We used the most recent such data available for each country if collected in or after 2014 (i.e, five years before the SRMNCHAH survey and reference year for MDG progress reporting) and imputed the World Bank income group mean for coverage in countries with only older data (Table S1 in the **Online Supplementary Document**). Due to the imperfect alignment between the timing of service utilization data across the 113 LMICs and the SRMNCAH policy survey, we selected an 85% cut-off point for deriving binary service utilization variables for our analyses.

### Statistical analyses

We fitted univariable logistic regression models for ≥85% coverage for each of four or more antenatal care visits (ANC4+), institutional delivery, PNC for the mother, and for all ANC4+, institutional delivery, and PNC for mothers, adjusting sequentially for each of the four maternal health policy categories and the full policy scores. This allowed us to examine the relative and overall importance of the four different domains of policy and policy structures. Lastly, we fitted multivariable logistic regression models for the same 4 outcomes adjusting for the full policy score and the contextual variables derived previously.

All analyses were conducted in Stata version 15 [26]. The 2018-2019 WHO SRMNCAH policy survey followed necessary WHO protocols for non-emergency, non-human-subject data collection. All data used in our analyses are publicly available (Table S1 in the **Online Supplementary Document**).

## RESULTS

In 2019, 84.1% of LMICs in our analysis had a coordinating body for SRMNCAH, but only 53.1% had a specific national technical working group for quality of care in maternal health (**Table 1**). A policy to improve quality of care with specific maternal health provisions was in place in 81.4% of LMICs and, importantly, quality of care standards and protocols specific to maternal health were available in 78.8% of these countries. Policies offering free ANC, uncomplicated vaginal deliveries, and PNC for the mother existed in over 80% of LMICs, with 77% of countries also offering cesarean sections and 79% offering management of pregnancy complications free of charge in public sector facilities. Most LMICs had national clinical guidelines for ANC (96.5%), childbirth (93.8%), and PNC (93.0%), yet a comprehensive set of counselling items were only available in about 40% of countries for ANC and childbirth and 55.8% of countries for PNC. A health provider competency framework for maternal and newborn health existed in 77.9% of LMICs, and 70.8% of countries had a continuous professional education system for such health care professional cadres. While 95.6% of LMICs had policies for birth registration, only about half have such policy for death registration. Notification of all maternal deaths to a central authority within 24 hours and reviews of all maternal deaths were mandated in 85.8% and 91.2% of LMICs, respectively, with 77.9% of countries calling for use of International Classification of Diseases – Maternal Mortality (ICD-MM) for classifying maternal deaths. Specific policies to conduct facility-based reviews of maternal deaths and to implement recommendations from maternal death reviews existed in 86.7% and 83.2% of countries, respectively, yet significantly less in upper-middle than low- and lower-middle income countries.

**Table 1:** Availability of Maternal Health Policies by World Bank income group in low- and middle-income countries, reported in percentages (n = 113)

Structure/policy/practice	Low-income group (n = 29)	Lower-middle income group (n = 40)	Upper-middle income group (n = 44)	*P*-value*	Total (n = 113)
**National supportive structures, and standards**					
Coordinating body for RMNCAH	86.2	80.0	86.4	0.682	84.1
QOC technical WG for MH	44.8	57.5	54.6	0.564	53.1
Policy to improve QOC with MH provisions	75.9	82.5	84.1	0.660	81.4
QOC standards and protocols specific for MH	75.9	80.0	79.6	0.905	78.8
Mean (SD) structure score†	2.8 (1.3)	3.0 (1.0)	3.0 (1.1)	0.758	3.0 (1.1)
**Service access policies in public sector**					
Human right to access MH services	44.8	62.5	70.5	0.087	61.1
Universal access to PHC	65.5	82.5	84.1	0.127	78.8
Free ANC services‡	75.9	85.0	95.5	0.050	86.7
Free normal childbirth services‡	72.4	77.5	88.6	0.192	80.5
Free cesareans‡	75.9	72.5	88.6	0.156	79.7
Free pregnancy complications management‡	72.4	67.5	88.6	0.057	77.0
Free PNC for women‡	72.4	82.5	93.2	0.057	84.1
Service access score, mean (SD)§	4.8 (1.9)	5.3 (2.0)	6.1 (1.7)	0.008	5.5 (1.9)
**National clinical guidelines & practices**					
Comprehensive clinical guidelines for ANC¶					
*Some counselling*	62.1	42.5	56.8	0.206	53.1
*Full counselling*	31.0	52.5	43.2		43.4
Comprehensive clinical guidelines for childbirth║					
*Some counselling*	55.2	50.0	54.6	0.700	53.1
*Full counselling*	37.9	46.5	36.4		40.7
Comprehensive clinical guidelines for PNC**					
*Some counselling*	37.9	32.5	40.9	0.800	37.2
*Full counselling*	51.7	62.5	52.3		55.8
Competency framework for MNH	75.9	80.0	77.3	0.913	77.9
Continuous professional education system in place for MNH providers	72.4	77.55	63.6	0.368	70.8
Education of midwifery care providers based on ICM competencies	79.3	65.0	47.7	0.022	62.0
Regulation of midwifery care providers based on ICM competencies	65.5	57.5	47.7	0.314	55.8
Clinical practice score, mean (SD)††	6.9 (2.2)	7.3 (2.0)	6.5 (2.2)	0.239	6.9 (2.2)
**National reporting and reviews**					
Registration of all births	93.1	95.0	97.7	0.627	95.6
Registration of all deaths	31.0	47.5	65.9	0.013	50.4
Notification to central authority of all maternal deaths within 24 h	93.1	90.0	77.3	0.106	85.8
Review of all maternal deaths	93.1	95.0	86.4	0.346	91.2
Maternal deaths classification using ICD-MM	72.4	85.0	75.0	0.388	77.9
Facility MDSR	86.2	97.5	77.3	0.024	86.7
Plan to implement MDR recommendations	93.1	90.0	70.5	0.015	83.2
Reporting score, mean (SD)‡‡	5.6 (1.4)	6.0 (1.2)	5.5 (2.2)	0.308	5.7 (1.7)
Total maternal health policy score, mean (SD)§§	20.1 (5.3)	21.6 (4.4)	21.2 (4.6)	0.476	21.1 (4.7)

ANC – antenatal care, ICM – International Confederation of Midwives, MDR – maternal death reviews, MDSR – maternal death surveillance and response, MH – maternal health, QOC – quality of care, PHC – primary health care, PNC – postnatal care, RMNCAH – reproductive, maternal, newborn, child, adolescent health, SBA – skilled birth attendance, WG – working group, SD – standard deviation

*χ^2^ tests for percentages and James’s test for equal means, allowing heterogeneous covariance matrices across by-groups.

†Summative score range 0-4.

‡Free services for all or selected population groups.

§Summative score range 0-7.

¶Specifies a minimum 4 number of contacts during normal pregnancy, timing of contacts with first occurring in the first 12 weeks of pregnancy, with specific statement on all of: birth preparedness and complication readiness, nutrition during pregnancy, iron and folic acid during pregnancy, immunization during pregnancy, screening for sexually transmitted infections, prevention and treatment of HIV, syphilis and TB in pregnancy, prevention and management of gestational diabetes, counselling on tobacco, alcohol, and substance abuse during pregnancy, partner involvement/ couple counselling, and intermittent preventive treatment in pregnancy (IPTp) for malaria in endemic countries.

║Specifies recommendation for the woman to choose the birthing position, availability of clean water, sanitation, essential equipment, and essential drugs, including magnesium sulfate for the prevention and treatment of eclampsia and any one of oxytocin, ergometrine or misoprostol for prevention and treatment of postpartum in the facilities where births take place.

**Specifies recommendations for the mother and baby rooming or being kept together until they are discharged from a facility; length of stay under observation of skilled attendant for mother and the baby, after normal childbirth, at facility; postnatal follow up contacts by a skilled attendant for mother and newborn after discharge from the facility; PNC contacts for both mother and newborn; minimum number of additional contacts after 24 h of birth within the first six weeks; describes who could provide care during the PNC contact(s) at home; and assessment of both mother and newborn at home.

††Summative score range 0-10, after scoring availability of “some counseling” with 1 and “comprehensive counseling” with 2 for the three clinical guidelines items.

‡‡Summative score range 0-7.

§§Summative score range 0-28.

The average maternal health policy scores for the four policy categories examined were 3 (score range = 0-4) for national supportive structures and standards, 5.5 (score range = 0-7) for service access in the public sector, 6.9 (score range = 0-10) for national clinical guidelines, and 5.7 (score range = 0-7) for national reporting systems, for an average total score of 21.1 (score range = 0-28) (**Table 1**). We found statistically significant differences by World Bank income group classification only for the service access policies category, with average scores of 4.8, 5.3, and 6.1 (score range = 0-7) for low, lower-middle, and upper-middle income countries, respectively (*P* = 0.008).

**Figure 2:**
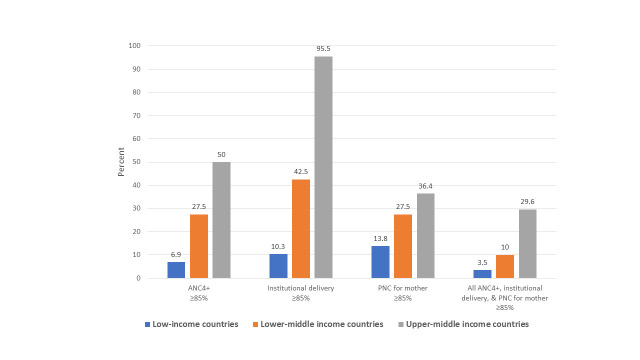
Maternal health service utilization outcomes by World Bank Income Group country categorization. Differences by World Bank income group are statistically significant at *P* < 0.05 or better for all outcomes except for PNC based on χ^2^ tests. ANC4+ – four or more antenatal care visits; PNC – postnatal care for the mother.

We documented the expected country variation in predictors of maternal health service utilization by World Bank income group, with the largest differences in the mean density of medical doctors per 10 000 population (22.7 in upper-middle income vs 4.2 in low-income countries) and mean domestic health expenditure per capita (575.9 vs 25.4 purchasing power parity (PPP), international US$; **Table 2**). Utilization of maternal health services also varied greatly by World Bank income group, with >85% coverage for all ANC4+ (*P* < 0.001), institutional delivery (*P* < 0.001), PNC for mother in only 3.5% of low-income (*P* = 0.107), 10% of lower-middle income, and 29.6% of upper-middle income countries (*P* = 0.005; **Figure 2**).

**Table 2:** Selected predictors of maternal health service utilization across WHO health system building blocks by World Bank income group in low- and middle-income countries (n = 113)

Indicators	Low-income group (n = 29)	Lower-middle income group (n = 40)	Upper-middle income group (n = 44)	Total (n = 113)
Governance*				
Political stability and absence of violence score	-1.1 (0.9)	-0.4 (0. 8)	-0.2 (0.7)	-0.5 (0.9)
**Health workforce**				
Density of medical doctors per 10 000 population, mean (SD)	4.2 (7.5)	7.8 (7.1)	22.7 (16.5)	12.7 (14.2)
**Health system financing**				
Domestic general government health expenditures per capita in PPP international US$, mean (SD)	25.4 (14.5)	136.6 (94.5)	575.9 (387.1)	279.1 (345.3)
**Contextual**				
Total fertility rate (number of children per woman)	4.6 (1.2)	3.2 (1.1)	2.7 (0.1)	3.2 (1.3)
Gender development index score, mean (SD)	0.8 (0.1)	0.9 (0.1)	1.0 (0.1)	0.9 (0.8)
Percent urban population, mean (SD)	36.2 (13.9)	46.8 (15.7)	64.9 (16.8)	51.1 (19.5)
Percent population with mobile cell phone subscriptions, mean (SD)	69.7 (33.9)	99.6 (29.2)	112.3 (36.5)	96.9 (37.2)

SD – standard deviation, PPP – purchasing power parity

*Governance indicators are reported in their standard normal units, ranging from approximately -2.5 to 2.5. Note the standard normal distribution is based on data from all countries in the Worldwide Governance Indicators database; therefore, the distribution of scores for the 113 low- and middle-income countries studied may not have a mean of 0 or SD of 1.

Higher maternal health policy scores, overall and for three policy categories (supportive structures and standards, service access in public sector, and national reporting systems and reviews), were associated with significantly higher odds of ANC4+ coverage≥85% and all ANC4+, institutional deliveries, and PNC coverage ≥85%, but not with PNC coverage ≥85% or institutional deliveries coverage ≥85% (**Table 3**). After adjusting for potential country context confounders, for each unit increase in the maternal health policy score, the odds of ANC4+≥85% increased by 37% (95% confidence interval (CI) = 1.13-1.64) and the odds of all ANC4+, institutional deliveries, and PNC≥85% by 31% (95% CI = 1.07-1.60). Among model covariates, the density of medical doctors was significantly associated with the odds of all ANC4+, institutional deliveries and PNC≥85% (odds ratio (OR) = 1.05; 95% CI = 1.01-1.10, *P* = 0.042), while the political stability score was only marginally associated with this outcome (OR = 2.84; 95% CI = 0.89-9.00, *P* = 0.077; data not shown).

**Table 3:** Relationships between maternal health policies and high maternal health service utilization in low- and middle-income countries (n = 113)

Maternal health policy categories	ANC4+≥85%, OR (95% CI)*	Institutional delivery, OR (95% CI)*	PNC for mother ≥85%, OR (95% CI)*	All ANC4+, institutional delivery and PNC for mother ≥85%, OR (95% CI)*
Score of national supportive structures, and standards	2.01 (1.25-3.21)‡	0.96 (0.69-1.34)	1.35 (0.90-2.03)	1.94 (1.03-3.63)‡
Score of service access policies in public sector	2.09 (1.37-3.18)‡	1.17 (0.96-1.43)	1.52 (1.10-2.10)‡	1.77 (1.07-2.92)‡
Score of national clinical guidelines and practices	1.17 (0.95-1.43)	0.89 (0.74-1.07)	1.05 (0.86-1.28)	1.09 (0.85-1.41)
Score of national reporting and reviews	1.53 (1.06-2.20)‡	1.05 (0.85-1.31)	1.08 (0.84-1.41)	2.18 (1.09-4.36)‡
Full policy score	1.29 (1.12-1.47)‡	1.01 (0.93-1.09)	1.11 (1.00-1.23)	1.25 (1.05-1.48)‡
Full policy score adjusted for covariates†	1.37 (1.13-1.64)‡	0.88 (0.74-1.04)	1.10 (0.98-1.25)	1.31 (1.07-1.60)‡

ref – reference, ANC4+ – four or more antenatal care visits, OR = odds ratio, CI – confident interval, OR – odds ratio, PNC – postnatal care for the mother

*<85% used as reference.

†Models adjusted for all variables shown and for political stability and absence of violence score, density of medical doctors per 10 000 population, domestic general government health expenditures per capita in purchasing power parity (PPP) international US$, total fertility rate, % urban population, gender development index score, % population with mobile cell phone subscriptions.

‡Statistically significant associations at *P* < 0.05 or better.

## DISCUSSION

LMICs need to strengthen their policy environments for maternal health. While supportive structures and standards, as well as free maternity service access policies, are in place and in line with international standards in most LMICs, we identified two key areas in great need of improvement: 1) clinical guidelines and practice regulations, and 2) national reporting and review systems for maternal mortality. The limited availability of national clinical guidelines for maternal health may indeed be (to a large extent) due to countries’ reliance on WHO guidelines, which conversely highlights the need for their regular updating and broad dissemination. Previous studies noted deficiencies in vital registration systems in LMICs [27,28], yet we document the extent to which death registration systems are lacking, especially in low and lower-middle income countries. Moreover, despite the apparent widespread availability of national policies recommending reporting, reviewing, and classifying maternal deaths at facility and national levels in LMICs, such data are not being contributed to WHO’s regular maternal mortality estimation exercises [7], raising questions about the actual adoption of these policies and the quality of reporting systems and data. Unarguably, having actionable data from maternal death reviews (i.e. patterns, causes, timing of maternal deaths) informs the design and implementation of maternal health programs and overall country investments to improve maternal health. Lessons learned from countries with established maternal death surveillance and response (MDSR) systems can provide valuable guidance on ways to set up functional identification and review processes at subnational and national levels [29]. For example, at the facility level, there is a need to establish review processes, employ a non-judgmental culture, offer the opportunity to reflect on on-going practices, and implement practice changes based on review recommendations. At the health system level, adequate funding and reliable health information systems are needed to enable identification and analysis of maternal deaths. At the national level, enforcement of mandatory notification of maternal deaths, a professional requirement to participate in maternal death reviews, and monitoring implementation of review recommendations were found to be useful strategies for MDSR systems to become embedded in the health system [29,30].

Favorable practice and policy contexts matter for maternal health. Our analysis found a significant, albeit modest association between countries having a more favorable policy environment for maternal health and high (≥85%) utilization of ANC, institutional delivery, and PNC services for the mother in 113 LMICs. This finding is in line with previous studies showing that country governance and policy are associated with improvements in the coverage of health interventions [31] and in mortality reductions [3,32,33]. However, the mere availability of policies and related structures is not sufficient to improve maternal health outcomes. An enabling policy environment will facilitate the scale-up of evidence-based interventions [34] but does not guarantee widespread coverage of these interventions. We found that the utilization of maternal health services in health facilities differs greatly across LMICs, despite >70% of these countries having policies making maternal health services free in public sector facilities. Research has shown that such policies are accompanied by higher service utilization soon after implementation, reducing the financial burden on the households and potentially contributing to a decline in inequity between the rich and poor [35,36]. Yet, it is well recognized that health facilities need to consistently offer high quality care to sustainably increase demand for and use of these services [37]. Consequently, recent research in LMICs identified the need to ensure facility readiness [38], target health professionals with training and other strategies to implement evidence-based care [39], and meet standards for respectful maternity care [40]. Aligning health care professionals’ education and practice regulations with evidence-based clinical guidelines is key to addressing these gaps. However, as shown in our analysis, these regulations and guidelines are lacking in 40%-45% of LMICs. Future research should explore what features of training and quality improvement interventions are effective in LMICs contexts and how they could be translated into another context.

Our study has some limitations. First, the list of policies and related structures we assessed is not comprehensive, and their selection and grouping were only guided by data availability and our expectation of the effects they may have on each of coordination, service access, clinical practices, and outcome reporting. Similarly, there are many contextual variables that influence policies and the functioning of health systems in LMICs [41] that we did not include in the analysis because we were limited by the availability of such variables across the 113 LMICs. Future research should consider larger sets of country policy and context variables, as well as how policies change and evolve over time, especially the shifts in global agenda for health information systems, birth and death registration, MDSR, and the usability and interoperability of these systems. A second limitation is that the availability of policies and related structures does not equate to adoption, functionality, and adaptability, or not equally across all LMICs in our analysis. Third, there is imperfect alignment between the timing of the cross-sectional (2018-2019) SRMNCAH survey and that of health service utilization data available for the various LMICs; this may have biased the association between maternal health service utilization and the policy environment for maternal health in either direction, despite our using a relatively high 85% cut-off point for assessing service utilization. Notably, the quality of data in our analysis likely varies by country and data source. SRMNCAH survey data are self-reported by country teams, mostly by representatives from the Ministry of Health or in-country offices of UN agencies [17]. We conducted a validation exercise to verify the information against country laws, policies, and guidelines, but we only used publicly available data. Data on contextual variables and outcomes from other data sources also vary in quality and reporting year. However, we collected these data using a standardized methodology and compiled from reliable data sources. Also, our analytical approach considered these data limitations and alignment with the timing of the SRMNCAH policy survey.

## CONCLUSIONS

This study presents a comprehensive review of the maternal health policy landscape in LMICs. Despite the availability of supportive structures and free maternity service access policies in these countries, there is a dire need for stronger policy support for clinical guidelines and practice regulations as well as national reporting and review systems for maternal mortality. A more favorable policy environment for maternal health can improve adoption of evidence-based interventions and increase utilization of maternal health services in LMICs.

## Additional material


Online Supplementary Document
