# Supplementary material for: Maternal health policy environment and the relationship with service utilization in low- and middle-income countries
Source: J Glob Health. 2023 May 10;13:04025. doi: 10.7189/jogh.13.04025 (PMC9997690; doi:10.7189/jogh.13.04025)
Supplement: Online Supplementary Document [file jogh-13-04025-s001.pdf]

ONLINE SUPPLEMENTARY DOCUMENT

Title: Maternal Health Policy Environment and the Relationship with Service Utilization in Low- and Middle-Income Countries

Authors: Andreea A Creanga, Martin AJ Dohlsten, Elizabeth K Stierman, Allisyn C Moran, Meighan Mary, Elizabeth Katwan, Blerta Maliqi

Table S1. Data sources and corresponding data years for predictors and maternal health service utilization data

Categories	Indicators	Data Sources	Years of data used in analyses
Governance	Political stability and absence of violence score	Worldwide Governance Indicators project[1]	2019 data available for 101 countries & imputation of income group mean for 12 countries
Health workforce	Density of medical doctors per 10,000	WHO Global Health Workforce Statistics database[2]	2019 data available for 27 countries; most recent available 2010-2018 data used for 99 countries; & imputation of income group mean for 14 countries
Health system financing	Domestic general government health expenditures, per capita in PPP international $	WHO Global Health Expenditure Database[3]	2018 data available for 105 countries & imputation of income group mean for 8 countries
Contextual	Total fertility rate<br>Urban population	Population Reference Bureau[4]	2020 for all 113 countries
	Gender development index, female-male ratio of human development index values (considers all education, life expectancy, GNI per capita)	United Nations Development Program[5]	2019 data available for 101 countries & imputation of income group mean for 11 countries
	Mobile cell phone subscriptions per 100 people	International Telecommunication Union World Telecommunication/ ICT Indicators Database[6]	2019 data available for 90 countries; 2018 data used for 12 countries; 2017 data used for 11 countries
Service utilization		UNICEF-compiled delivery care data[7]	We used the most recent coverage data available for each country if collected in or after 2014 and imputed the income group mean for coverage in countries with older data.

	Antenatal care (4+ visits)		2014-2020 Antenatal care 4+ data available for 82 countries & imputation of income group mean for 31 countries.
	Institutional deliveries		2014-2020 institutional delivery data available for 86 countries & imputation of income group mean for 27 countries.
	Postnatal care for the mother		2014-2020 Postnatal care data available for 75 countries & imputation of income group mean for 38 countries.
	Caesarean delivery		2014-2020 caesarean data available for 71 countries & imputation of income group mean for 42 countries

GNI – Gross national income, ICT – Information and communications technology, PPP – purchasing power parity.

REFERENCES

1. World Bank Group. Worldwide Governance Indicators. 2021 update [Internet]. 2022. Available at: http://info.worldbank.org/governance/wgi/.

2. World Health Organization. Global Health Workforce statistics database. 2022. Available from: https://www.who.int/data/gho/data/themes/topics/health-workforce.

3. World Health Organization. Global Health Expenditure Database Geneva. 2022. Available at: https://apps.who.int/nha/database.

4. Population Reference Bureau. 2019 World Population Data Sheet. 2020. Available at: https://www.prb.org/resources/2019-world-population-data-sheet/

5. World Bank Group. World Development Indicators. 2022. Available at: https://datatopics.worldbank.org/world-development-indicators/

6. World Telecommunication/ICT Indicators Database. 2022. Available at: https://www.itu.int/en/ITU-D/Statistics/Pages/publications/wtid.aspx

7. UNICEF. Delivery care data. 2022. Available at: https://data.unicef.org/resources/dataset/delivery-care/
